# Real-World Insights in Designing SteatoStat: An End-to-End Deep Learning Pipeline for Hepatic Steatosis Quantification

**DOI:** 10.3390/diagnostics16121825

**Published:** 2026-06-12

**Authors:** Nagalakshmi Jegannathan, Xiaoman Zhang, Jia Xuan Seow, Menghan Zhou, Long Wang, Guo Lin Goh, Seow Ye Heng, Tony De Rong Ng, Rick Siow Mong Goh, Huazhu Fu, Yong Liu, Lionel Tim-Ee Cheng, George Boon Bee Goh, Dean Tai, Chee Leong Cheng, Wei Keat Wan, Tony Kiat Hon Lim, Li Yan Khor, Wei Qiang Leow

**Affiliations:** 1 Department of Anatomical Pathology, Singapore General Hospital, Singapore 169856, Singapore; nagalakshmi.jegannathan@sgh.com.sg (N.J.); heng.seow.ye@sgh.com.sg (S.Y.H.); cheng.chee.leong@singhealth.com.sg (C.L.C.); lim.kiat.hon@singhealth.com.sg (T.K.H.L.); khor.li.yan@singhealth.com.sg (L.Y.K.); 2 Institute of High-Performance Computing, Agency for Science, Technology and Research, Singapore 138632, Singaporezhou_menghan@ihpc.a-star.edu.sg (M.Z.); wangl@ihpc.a-star.edu.sg (L.W.); gohsm@ihpc.a-star.edu.sg (R.S.M.G.); fu_huazhu@ihpc.a-star.edu.sg (H.F.); liuyong@ihpc.a-star.edu.sg (Y.L.); 3 School of Biological Sciences, Nanyang Technological University, Singapore 637551, Singapore; gohg0010@e.nut.edu.sg; 4 Ministry of Health Holdings, Singapore 139691, Singapore; de.rong.ng@mohh.com.sg; 5 Department of Cardiothoracic and Abdominal Radiology, Singapore General Hospital, Singapore 169608, Singapore; lionel.cheng.t.e@singhealth.com.sg; 6 Duke-NUS Medical School, Singapore 169857, Singapore; 7 Department of Gastroenterology and Hepatology, Singapore General Hospital, Singapore 169608, Singapore; goh.boon.bee@singhealth.com.sg; 8 HistoIndex Pte., Ltd., Singapore 117674, Singapore; dean.tai@histoindex.com

**Keywords:** deep learning pipeline, hepatic steatosis, MASLD, segmentation model

## Abstract

**Background**: Metabolic dysfunction-associated steatotic liver disease (MASLD) is a significant and escalating global health concern, with an estimated prevalence of 30%. Current assessments of hepatic steatosis, a hallmark of MASLD, rely on semi-quantitative grading by pathologists, which is inherently limited by inter-observer variability. **Objective**: To address this limitation, we developed a novel deep learning pipeline, named SteatoStat, to standardize and enhance the quantification of hepatic steatosis in patients with MASLD. **Method:** The SteatoStat pipeline employs and integrates multiple components such as file format standardization, rule-based cell filtering, and multiple segmentation models across various liver structures, resulting in an output of a continuous quantitative measure of steatosis percentage and translated into steatosis grades. **Results**: We report a high degree of accuracy and reliability with SteatoStat achieving the following performance metrics (DICE score = 0.8955, AUROC = 0.9928, F1 score = 0.8990). When benchmarked against expert pathologists, the weighted Kappa coefficient is 0.837. Furthermore, in comparison with an existing, well-established model, SteatoStat demonstrated a weighted Kappa coefficient = 0.765. **Conclusions**: These robust findings underscore its potential clinical utility in providing a standardized objective quantification of hepatic steatosis. Future directions include enhancing the model’s generalizability and its clinical integration through validation on independent, multi-institutional datasets.

## 1. Introduction

Metabolic syndrome (MetS) is characterized by the components of abdominal obesity, atherogenic dyslipidemia, raised blood pressure, insulin resistance with or without glucose intolerance, a proinflammatory state and a prothrombotic state, which are significant risk factors for the development of cardiovascular disease (CVD) and type II diabetes mellitus [1].

Non-alcoholic fatty liver disease (NAFLD), the hepatic manifestation of metabolic syndrome (MetS), is associated with hypertriglyceridemia, low HDL, hypertension, and obesity [2]. It is defined by ≥5% hepatocyte steatosis without secondary causes (e.g., alcohol or drugs) [3]. NAFLD includes simple steatosis [4] and its progressive form, non-alcoholic steatohepatitis (NASH), which may lead to cirrhosis, liver failure, or hepatocellular carcinoma [3].

To reduce stigma, ~60% of experts in a Delphi consensus supported adopting steatotic liver disease (SLD) to encompass all causes of steatosis. The subtype metabolic dysfunction-associated steatotic liver disease (MASLD) replaces NAFLD and is defined by steatosis with ≥1 MetS component [5]. MASLD is the most common chronic liver disease and a leading cause of liver-related morbidity and mortality [6]. The global prevalence of MASLD is estimated to be around 30%, with its disease burden expected to increase over time [7]. This emphasizes the importance of continued research in MASLD to understand the disease as well as to find therapeutic targets. Hence, there have been many studies done on the different components of MASLD, such as steatosis, inflammation, ballooning and fibrosis, to be able to grade or stage the disease accurately and chart the progression of the disease in patients. Even though studies have shown that hepatic fibrosis is the only independent predictor of clinical disease progression [8], there are also other studies which focus on the other components of MASLD such as steatosis [9], lobular inflammation [10] and ballooning [11].

MASLD diagnosis requires either radiographic or histological identification of ≥5% hepatic steatosis in the absence of alcohol consumption [12]. According to the American Association for the Study of Liver Diseases’ (AASLD) guidelines, a conclusive diagnosis of NASH requires a liver biopsy with histologic analysis identifying hepatocyte ballooning degeneration, hepatic lobular inflammation, and ≥5% hepatic steatosis [13], as liver biopsy is the gold standard for quantifying and staging MASLD [14].

At present, pathologists give a semi-quantitative analysis of fat percentage (steatosis) by reviewing the liver biopsy section that is stained with the Hematoxylin and Eosin (H&E) stain. While grading, lobular inflammation and presence of balloon cells are also considered, along with fat percentage. The current grading system, which is being used to grade and stage MASLD, is the NASH Clinical Research Network (NASH CRN) scoring system as shown in Table 1 [15,16].

Table 1 summarizes the NASH Clinical Research Network (NASH CRN) scoring system, which categorizes steatosis based on defined percentage thresholds, together with assessments of lobular inflammation, ballooning, and fibrosis. While this remains widely used, alternative systems such as the Steatosis–Activity–Fibrosis (SAF) score have also been proposed, highlighting the semi-quantitative nature of current grading frameworks.

Furthermore, conventional steatosis scoring is relatively imprecise [4], making it difficult to detect subtle disease progression. In contrast, artificial intelligence (AI)-based approaches enable fat content to be quantified on a continuous scale, allowing for more precise assessment and improved sensitivity in tracking disease progression [17]. A previous study by HistoIndex has shown the quantification of hepatic steatosis in chronic liver disease using an automated learning machine-based method, following second harmonic generation (SHG) and two-photon excited fluorescence (TPEF) microscopy [18]. The results show an excellent correlation between the histopathologists’ assessment and the severity of steatosis calculated by the SHG method. However, SHG/TPEF is very costly and has limited applicability, which makes it impractical to implement in clinical practice [11]. Furthermore, this study requires additional steps as it uses unstained slides which are usually not part of the clinical routine. Hence, we have chosen to use an AI software that analyses routine H&E-stained samples so that it can be better incorporated into most clinical laboratory workflows.

**Table 1:** NASH CRN scoring system. Defines the specific percentage thresholds (<5%, 5–33%, 34–66%, >66%) used to assign steatosis scores (0 to 3), along with lobular inflammation, ballooning and fibrosis during the histopathological assessment of NAFLD/MASLD [16,19].

	Score/Grade	Description
Steatosis	0	<5%
	1	5–33%
	2	>33–67%
	3	>67%
Lobular Inflammation	0	No foci
	1	<2 foci/20×
	2	2–4 foci/20×
	3	>4 foci/20×
Ballooning	0	No ballooning
	1	Few ballooned cells
	2	Many ballooned cells
Fibrosis	0	No fibrosis
	1	1a Mild, zone 3 perisinusoidal/pericellular fibrosis
		1b Moderate, zone 3 perisinusoidal/pericellular fibrosis
		1c Portal/periportal fibrosis
	2	Perisinusoidal/pericellular and portal/periportal fibrosis
	3	Bridging fibrosis
	4	Cirrhosis

Additionally, the current approach to assess a drug’s efficacy in reducing liver fat percentage is still subject to substantial refinement, highlighting the limited availability of a reliable and validated method during clinical trials. Currently, drug efficacy is primarily evaluated based on changes in steatosis grades, which represent a broad classification and may not fully capture smaller yet significant reductions in hepatic fat percentage. This approach presents a limitation, as it may cause drugs to be deemed ineffective despite improvements. This underlines the need for a more accurate and precise method for quantifying hepatic steatosis in order to better assess treatment outcomes. Therefore, there remains a significant and urgent need for a tool that can be reliably utilized by pathologists to assess and quantify liver fat, facilitating more accurate and precise measurements for accelerated drug approval [4].

Several recent studies have explored artificial intelligence-based approaches for hepatic steatosis quantification, including convolutional neural network-based segmentation models [20] and multi-stage pipelines incorporating graph-based or pixel-level analysis [21]. While these approaches have demonstrated promising performance, many are limited by reliance on single-scanner datasets, use of non-routine staining modalities, or focus on isolated components of the histological workflow. Furthermore, existing methods often lack integration across multiple tissue structures and do not explicitly address variability arising from multi-scanner and multi-format whole slide images. Consequently, there remains a need for a unified, end-to-end pipeline that is capable of robust performance across diverse real-world clinical data while maintaining interpretability and alignment with pathological assessment workflows.

Therefore, in this study, we aim to develop a robust end-to-end deep learning pipeline for hepatic steatosis to support the diagnosis and assessment of MASLD.

## 2. Materials and Methods

**Figure 1:**
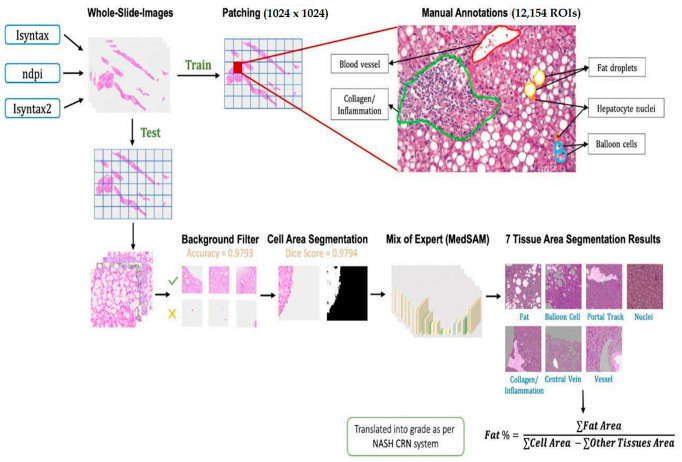
End-to-end deep learning liver tissue area segmentation pipeline.

We developed an end-to-end deep learning liver tissue area segmentation pipeline focused on quantification of hepatic steatosis, named SteatoStat (v1). The pipeline calculates the fat percentage by dividing the total fat area by the total cell area excluding the area occupied by balloon cells, portal tracts, central veins, blood vessels, collagen, and inflammation as shown in the formula in Figure 1.

### 2.1. Data Preprocessing

#### Sample Collection and Scanning

A total of 41 archived formalin-fixed, paraffin-embedded (FFPE) liver biopsy samples were included for whole slide imaging. These samples were obtained from patients who underwent liver biopsy for clinical indications between 2005 and 2017 and had a clinical diagnosis of NAFLD/MASLD. This study utilized a single-center retrospective cohort consisting strictly of Formalin-Fixed Paraffin-Embedded (FFPE) percutaneous core needle liver biopsy samples. To ensure the reliability of our findings amidst routine sampling variability, only specimens obtained via standard percutaneous needle biopsy were included; surgical wedge biopsies were excluded. The typical core length averaged 15–20 mm, containing a median of 10 portal tracts per sample. Suboptimal or highly fragmented specimens were managed by exclusion from the primary analysis. Patients with excessive alcohol consumption—defined as more than 21 units per week for males and more than 14 units per week for females—were excluded, resulting in a final sample size of 41.

The liver biopsy samples were then processed as per routine clinical practice and cut into 4 µm-thick sections before being stained with H&E. Subsequently, the slides underwent a blinded assessment by a team of three expert hepatopathologists who assessed the fat percentage and graded the slides based on the NASH CRN scoring system (Table 1). Discrepant cases were reviewed at a multi-header microscopy session for a consensus score. For agreement analysis, a subset of 20 cases was selected based on the availability of consensus pathologist annotations, which are required for reliable comparison with model predictions.

The Philips Ultra-Fast Scanner, Philips Pathology Scanner Second Generation (Koninklijke Philips N.V., Amsterdam, The Netherlands) and NanoZoomer S60 Digital slide scanner (Hamamatsu, Hamamatsu City, Japan) were used to scan glass slides into digital pathology Whole Slide Images (WSIs) as iSyntax, iSyntax2 and NDPI files, respectively.

### 2.2. Cell Patch Extraction

The files were then converted into BigTIFF format, and WSIs were cut into patches of 1024 by 1024 pixels as part of the pre-processing procedure. Patch-based processing is used in WSI segmentation because full-resolution whole-image inference is computationally infeasible. This approach remains a practical method for preserving fine-grained diagnostic details while maintaining manageable memory usage. Our method is therefore designed for high-resolution WSI segmentation under these practical constraints, rather than to claim any inherent superiority over whole-image strategies.

### 2.3. Tissue Area Annotation

The pre-processed images were uploaded onto the Open Microscopy Environment (OMERO) server, and 12154 regions of interest (ROIs) composed of seven different structures, such as the fat vacuoles, nuclei, blood vessels, portal tracts, central veins, balloon cells, collagen and inflammation, were annotated for training and validation.

### 2.4. Pipeline Architecture

#### 2.4.1. Background Filter

**Figure 2:**
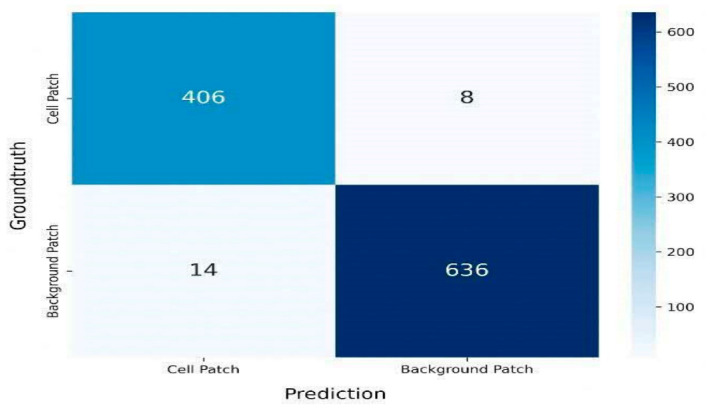
Confusion matrix of background filter model on test images.

The whole slide images are divided into patches of 1024 by 1024 pixels. We trained a multilayer perceptron (MLP) model on 2037 images and tested it on 1064 image samples using a single train–test split at the image-level. We employed the trained background filter model to identify tissue material. The test accuracy of the background filter model is 0.9793 (confusion matrix shown in Figure 2). The high performance of the model effectively filters out the background noise, ensuring that subsequent analyses focus on relevant tissue material data without the interference of irrelevant artifacts.

#### 2.4.2. Cell Area Segmentation

The cell area segmentation module utilizes a U-Net architecture, renowned for its effectiveness in biomedical image segmentation. The U-Net model is trained on 356 images with manual annotations and tested on 87 samples. The DICE score of the test dataset is 0.9794. This model segments cellular regions within the patches, facilitating detailed analysis of cellular structures and morphologies.

#### 2.4.3. Modular Integration U-Net and SAM

To address the segmentation of the aforementioned structures, we initially employed U-Net, a model specifically designed for biomedical image segmentation. U-Net features an encoder path, which facilitates the extraction of hierarchical features, and a decoder path, which reconstructs the segmented output [22]. Despite the limitations of our dataset, U-Net demonstrated its capacity for efficient training [23], achieving precise object localization and classification during segmentation. Additionally, its compatibility with average hardware makes it a practical choice for diverse applications [24]. Specifically, rather than fully fine-tuning all the SAM parameters, we adopted a parameter-efficient adaptation strategy as in Medical SAM Adapter (Med-SAM). We froze the original SAM components (image encoder, prompt encoder, and mask decoder) and inserted lightweight Adapter modules into the transformer blocks, training only the adapter parameters [25].

SAM has been pre-trained on extensive datasets, enabling robust segmentation across a wide range of images. By leveraging this pre-trained foundation model, the need for large volumes of annotated pathology data is significantly reduced. This approach allows the model to effectively address the unique characteristics of medical images, including the integration of pathology-specific details that are essential for accurate and reliable medical image analysis. The raw fat percentage is calculated using pixel quantification according to the formula provided. However, discrepancies have been observed between this method and the fat percentage assigned by the pathologist, which reflects fat content as part of the NAFLD activity score. The segmentation-based approach enables explicit quantification of fat percentage at the pixel level, which is subsequently mapped to clinically relevant steatosis grades using the calibration model. This design provides interpretability and transparency by allowing direct visualization and measurement of fat regions, aligning with standard pathological assessment workflows. In contrast to end-to-end classification models, which directly predict grades without intermediate representation, this approach preserves explainability and facilitates validation at each stage of the pipeline. To address this discrepancy, a nonlinear mapping model was developed. The calibration model was implemented as a multilayer perceptron (MLP)-based regression model, which learns the relationship between pixel-quantified fat percentage (at the WSI level) and pathologist-assigned scores as input. Mean Squared Error (MSE) loss is utilized to optimize the model and refine the predictions in accordance with the NAFLD activity score. To ensure robustness and prevent data leakage, WSIs were split at the patient level prior to patch extraction, with no overlap between training, validation, and test sets.

#### 2.4.4. Application

**Figure 3:**
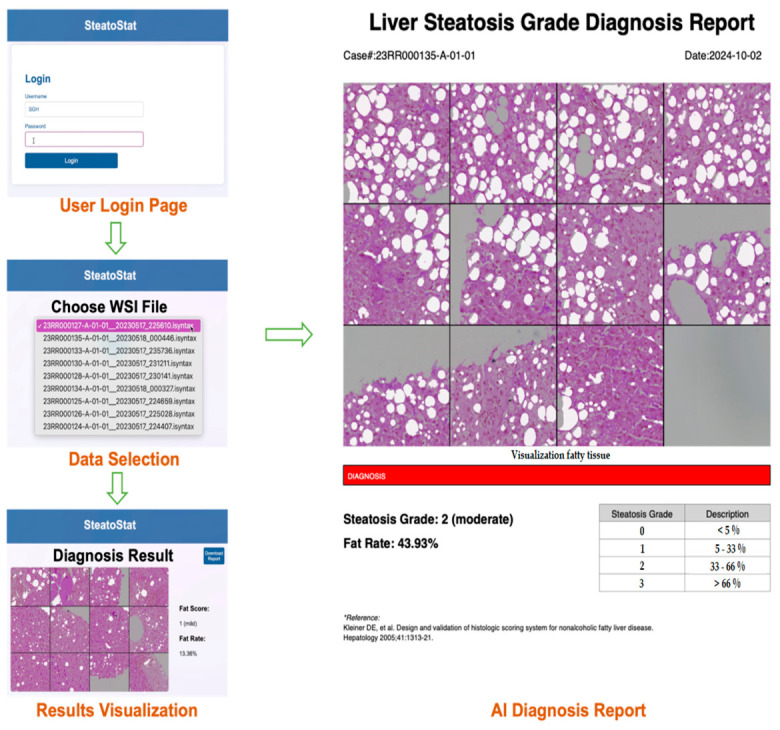
User interface workflow with final report. * Steatosis grading thresholds are derived from the histological scoring system proposed by Kleiner et al. (2005) [16].

We developed a robust front-end and back-end pipeline for liver pathology imaging. The back-end pipeline user interface workflow is shown in Figure 3.

A secure, user-authenticated software interface facilitates the selection of uploaded WSIs for analysis. SteatoStat autonomously performs quantitative analysis of hepatic steatosis. The resulting output is an easy-to-read report, featuring a visual representation of the steatosis-affected regions alongside the calculated hepatic steatosis percentage and corresponding NASH-CRN steatosis grade.

### 2.5. Experiment

#### 2.5.1. Experiment Setting and Implementation Details

We employed a 5-fold cross-validation strategy to evaluate the performance of our model. The patch dataset was randomly split into five folds. In each iteration, one fold served as the test set, one as the validation set, and the remaining three as the training set, ensuring every fold was used for testing and validation. The data split was conducted at the whole-slide image (WSI) level prior to patch extraction, rather than at the ROI or patch level. As each WSI in the dataset corresponds to a unique patient, this WSI-level division effectively functions as a patient-level split. Accordingly, all the patches derived from a given WSI were assigned exclusively to either the training, validation, or test set, thereby preventing any possibility of data leakage. The non-integer validation proportion arises because different WSIs generate varying numbers of patches after tiling. Since the split is defined at the WSI/patient level—rather than by enforcing a fixed patch ratio—the resulting patch percentages naturally do not align to exact integers. This characteristic is an expected consequence of patient-level splitting and does not reflect any irregularity in the allocation procedure. The experiments were conducted on an NVIDIA GeForce RTX 3090 (Santa Clara, CA, USA). The learning rate was adjusted within the range of 0.0001 to 0.01, with a batch size of 2.

The calibration model was applied as a post hoc mapping at the WSI level and was not incorporated within the cross-validation framework; therefore, calibration parameters were not independently estimated and evaluated across separate data splits

#### 2.5.2. Evaluation Criterion

To assess the performance of the segmentation model, we utilized three key metrics: DICE score, F1 score, and Area Under the Receiver Operating Characteristic Curve (AUROC).

DICE score quantifies the overlap between the predicted and ground truth masks. It is calculated as DICE score = 2 × ∣P ∩ G∣∣P∣ + ∣G∣ where P represents the predicted segmentation, and G represents the ground truth. A higher Dice score indicates better segmentation performance. The DICE score is calculated using the formula below:

Sorenson-DICE Index = (2 × number of true positives)/(2 × number of true positives + number of false positives + number of false negatives)(1)

F1 score represents the harmonic mean of precision and recall; the F1 score evaluates the balance between false positives and false negatives. F1 is calculated using the formula below:

F1 = 2 × Precision × Recall/(Precision + Recall).(2)

This metric is particularly useful for imbalanced datasets.

AUROC: The AUROC evaluates the model’s ability to distinguish between classes across various threshold settings. It measures the area under the ROC curve, which plots the true positive rate against the false positive rate. A higher AUROC value reflects better discrimination capability.

## 3. Results

### 3.1. DICE Score

**Figure 4:**
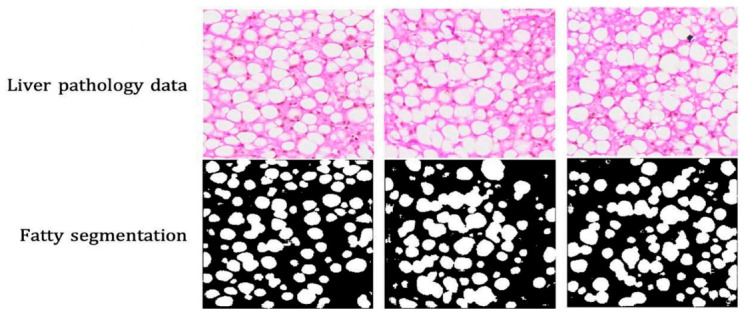
Visualization of fat segmentation results against biopsy tissue.

SteatoStat, under the U-Net segmentation, achieved a DICE score of 0.8955 when trained and tested on a combination of WSI file formats. Table 2 shows the varying DICE scores when trained and tested on different WSI file formats. The DICE score was the lowest) for NDPI and highest when a combination of different WSI file formats was used. The analysis of individual WSI file formats was conducted using the U-Net model to evaluate format-specific variability, while SAM was evaluated under a combined multi-format setting and is therefore not included in Table 2. Figure 4 shows a visual representation of matching biopsy tissue and segmentation patches.

During the initial analysis, we obtained a high DICE score for fat segmentation, although the DICE scores for the other anatomical structures were comparatively lower. Table 3a shows the comparison scores for fat and other liver structures using U-Net. After applying 5-fold cross-validation to both models, the DICE score for fat decreased in U-Net, whereas SAM maintained a consistently high level of performance. The segmentation performance for the remaining liver structures continued to vary across both models.

### 3.2. AUROC and F1

SAM outperformed U-Net in classification tasks, achieving higher AUROC and F1 scores, indicating improved discrimination between fat and non-fat regions (Table 4).

Performance metrics, including AUROC, F1 score, precision, and recall, for SAM and U-Net segmentation models following 5-fold cross-validation are summarized in Table S1 a and b respectively.

### 3.3. Correlation Analyses Against Pathologists

SteatoStat showed a weighted Cohen’s Kappa coefficient of 0.837 when the predicted steatosis grade was compared against the pathologists’ grade. SteatoStat correctly predicted the steatosis grade in 17 out of 20 cases (subset used for agreement analysis). Of the three wrong predictions, there was one instance of underestimation and two instances of overestimation (Table S2).

### 3.4. Correlation Analyses Against Existing Well-Established Model

**Figure 5:**
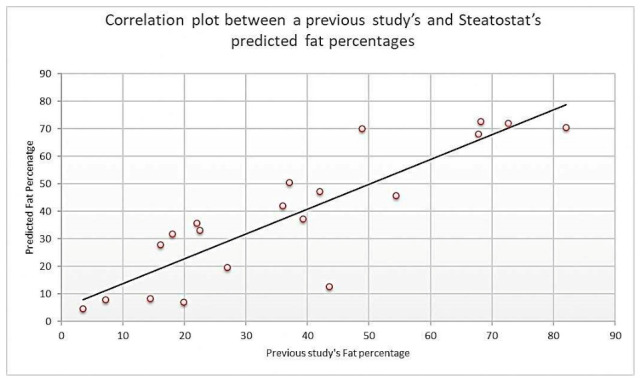
Strong correlation (r = 0.86) observed in the Pearson plot between HistoIndex study’s and SteatoStat’s predicted fat percentages.

A quantitative comparison between SteatoStat and the previously established HistoIndex model [17] demonstrates strong agreement across multiple evaluation metrics. SteatoStat achieved a weighted Cohen’s Kappa coefficient of 0.765 when compared with the HistoIndex model, indicating substantial agreement. In addition, a strong positive correlation (r = 0.86) was observed between the predicted fat percentages of the two models (Figure 5). In terms of classification agreement, SteatoStat correctly matched the HistoIndex-derived steatosis grade in 18 out of 20 cases (same subset used for agreement analysis with pathologists), with only two discordant cases (Table S3). These results provide a clear quantitative assessment of agreement between the two approaches and should be interpreted as internal validation.

## 4. Discussion

Previous studies have shown the use of AI technology to diagnose MASLD through different methods.

A recent study about the clinical validation of an AI-based pathology tool for scoring of metabolic dysfunction-associated steatohepatitis (MASH) developed and trained their AIM-MASH model using convolutional neural networks (CNN). These networks utilized de-identified WSIs of liver biopsies that had been annotated after staining with either H&E or Masson’s trichrome. The model then incorporated graph neural networks (GNNs) to calculate the area and predict grades or stages of steatosis, lobular inflammation, hepatocellular ballooning, fibrosis, and stain-induced artifacts. The AIM-MASH model demonstrated a strong capability of correctly identifying steatosis, achieving an accuracy of approximately 0.96 (95% CI, 0.93–0.98) [20].

Another study focused on the quantification of the various MASLD-related features using a machine learning approach. When compared to our study, this one incorporated an additional Sirius red stain. Moreover, it used a single scanner, the Hamamatsu whole slide scanner (Shizuoka, Japan), exporting images into JPEG format for analysis and quantification. The AI pipeline involved a four-stage process assessing steatosis, inflammation and ballooning in relation to the biopsy core. The study calculated the fat percentage based on the pixel color analysis, yielding median values of 2.6% (interquartile range [IQR], 1.7–3.8%) for grade 1, 15.1% (IQR, 10.1–20.1%) for grade 2 and 28.4% (IQR, 20.2–31.9%) for grade 3 [21].

Additionally, a study on the automated segmentation and morphological characterization of hepatic steatosis utilized previously published, well-characterized archived histological samples from 72 liver transplants. These patients were followed up at one-year post transplant as per their center protocol. The liver biopsy samples were obtained from the transplantation, and an additional Masson’s trichrome stain was used when compared to our study. The WSIs were then analyzed to compute fat percentage, with fat-affected hepatocyte ratio (FHR) emerging as a key metric, showing strong correlation with the pathologist’s Clinical Research Network (CRN) fat grading. However, a key limitation is the reliance on a single pathologist for histopathological grading and visual validation of segmentation results without phantom images, which could potentially introduce bias [26].

Furthermore, another study investigated deep learning-based hepatic steatosis quantification of liver biopsy samples, which were processed similarly to our study. Their methodology involved cutting the WSIs into patches and extracting them at 20× objective magnification using the OpenSlide software (v1.1.1). The model employed a combination of image segmentation models to identify different regions and boundaries within the tissue. While high accuracy and specificity were reported, limitations included potential under-representation due to the random patch and the challenge of analyzing inherently three-dimensional droplets on two-dimensional slides, potentially introducing measurement error [9].

Collectively, these studies highlight the growing utility of AI in the quantification and grading of MASLD-related histological features. Despite the extensive body of research on hepatic steatosis quantification, it is evident that multiple methodologies are being employed, each with unique advantages and limitations. This underscores the need for a more standardized AI-driven pathology framework that can ensure consistency, accuracy and reproducibility across different clinical and research settings.

**Comparison of SteatoStat and Expert Pathologists**

SteatoStat was able to achieve a stably high DICE score of 0.8955 (U-Net) and 0.8542 (SAM), regardless of whether U-Net or SAM was utilized in the pipeline (Table 2). The high DICE scores indicate a high degree of concordance between the predictions and ground truth, highlighting SteatoStat’s accuracy in segmentation of hepatic steatosis.

Further supporting SteatoStat’s strong performance are its high AUROC (0.9928) and F1 score (0.8990), reflecting excellent capability in distinguishing fat from non-fat areas. The high F1 score underscores a reliable classification by minimizing under-diagnosis in patients with significant steatosis as well as over-diagnosis in those with minimal steatosis, thereby minimizing the risk of diagnostic errors.

In addition, the predicted grades show excellent agreement with the pathologists’ grades, reflected by a Cohen’s Kappa coefficient of 0.837, which underscores the model’s robustness and consistency with the ground truth.

There were three cases (out of 20) that were either underestimated or overestimated (Table S2; Figure S3).

Instances of underestimation were primarily due to the model’s limited ability to accurately segment micro vesicular steatotic areas. While the MLP-based calibration module effectively minimized residuals in high-fat samples, it may have introduced overestimation in lower-grade steatosis by adjusting predictions upward to align with the learned regression trend.

When benchmarked against the pathologists’ estimates, SteatoStat’s predicted fat percentage showed a strong correlation (r = 0.92; Figure S1) with a lack of statistically significant deviation (*p* = 0.98; Figure S8). This highlights that SteatoStat predictions track closely with actual fat percentages.

**Comparison of SteatoStat and Histoindex**

In our previous collaborations, we worked with Histoindex Pte Ltd. to quantify hepatic steatosis. The previously reported HistoIndex model demonstrated strong correlation with NASH-CRN scoring (r = 0.802) and AUROC values ranging from 0.939 to 0.986, providing a benchmark for comparison with SteatoStat.

In comparison, SteatoStat achieved a higher correlation (r = 0.86) and demonstrated substantial agreement (Cohen’s Kappa = 0.765), supporting its comparable performance relative to the HistoIndex model [17].

SteatoStat demonstrated good discriminatory ability (Figure S5) across the full range of steatosis grades (Table S3). Only 2 cases (out of 20) were discordant.

SteatoStat exhibited sub-optimal segmentation in a case with extensive areas of micro vesicular steatosis, possibly due to the subtle and diffuse nature of the fat droplets. Over-segmentation was observed in the other case with predominance of macro vesicular steatosis, possibly from misidentifying similar clear spaces. When standardized against a previously established model, SteatoStat demonstrated a strong positive correlation (r = 0.86; Figure 5) and no significant bias (Figure S4), indicating consistent performance across both models.

Overall, these findings demonstrate that SteatoStat is a consistent and reliable tool for hepatic steatosis quantification in both clinical and research settings.

**Real-World Issues and Insights**
**Multiple Scanners, Multiple File Formats**

The observed variability in model performance across file formats is hypothesized to be attributable to domain shift. To improve domain robustness, the training dataset was expanded to include whole-slide images (WSIs) from three file formats (iSyntax, iSyntax2, and NDPI). Under this multi-format training setting, the mean DICE score for fat segmentation increased to 0.8955 (Table 2).

Data partitioning was performed at the WSI level prior to preprocessing and patch extraction, with each WSI corresponding to a unique patient identifier. Consequently, all the patches derived from a given WSI were assigned exclusively to a single subset (training, validation, or test), ensuring the absence of overlap or data leakage between partitions.

Inclusion of WSIs acquired from multiple scanners and file formats, following normalization, increased dataset diversity and improved robustness to inter-scanner variability. However, this improvement should not be interpreted as evidence of formal domain adaptation. Rather, the performance gain more appropriately reflects enhanced generalizability under multi-scanner, multi-format conditions, independent of any overlap between data subsets.

This approach confers practical advantages in real-world clinical settings, as the pipeline operates directly on routine H&E-stained slides from different scanners and heterogeneous file formats without requiring specialized staining or dedicated imaging platforms. The incorporation of data format standardization and multi-scanner training enables robustness to domain shifts commonly encountered in pathology workflows, thereby enhancing scalability and facilitating integration into existing digital pathology infrastructures across institutions.

**Overcoming a Small Dataset**

Aside from integrating various algorithms such as background filter and cell area segmentation into the pipeline to enhance the pipeline, we explored and compared the use of SAM, a pre-trained foundation model, against the typically used U-Net. As training a model from scratch typically requires a large dataset to achieve optimal performance, fine-tuning a foundation model such as SAM allowed us to overcome the limitation of a small dataset. This can be seen by the increase in AUROC (0.9928) and F1 values (0.8990) (Table 4), reflecting that SteatoStat is well-suited for real-world variability.

**Tackling Performance in High Steatosis Samples**

In the initial testing of our cohort, we noticed that SteatoStat frequently underestimates hepatic steatosis relative to pathologists’ assessments. We postulate that this systematic discrepancy may be arising because pathologists visually estimate the proportion of hepatocyte cells containing lipid droplets rather than the absolute area occupied by the lipid droplets. Additionally, H&E staining dissolves many small lipid droplets, leaving behind small clear vacuoles that are often missed by simple thresholding techniques. To address this systemic under-estimation, particularly in high-grade steatotic samples, we implemented a post hoc regression-calibration module using a multilayer perceptron (MLP). The MLP learns a data-driven mapping from the raw fat-pixel-to-cell-area ratio to the visually assessed steatosis grade. By minimizing residuals on a held-out training subset, the network recalibrates the initial pixel-based estimates toward pathologist-derived percentages. This adjustment significantly reduces bias and restores accuracy in the high steatosis range, where conventional segmentation approaches tend to plateau.

**Tissue Area Segmentation and Annotations**

Aside from annotating fat, we have also annotated other liver structures as shown in Figure 1. Our annotations could serve as a stepping stone for follow-on research, providing a framework that can be expanded and refined to explore new methodologies and applications within this domain, such as quantification of fibrosis, ballooned hepatocytic cells and inflammation.

**Integration into Laboratory Clinical Workflow**

**Figure 6:**
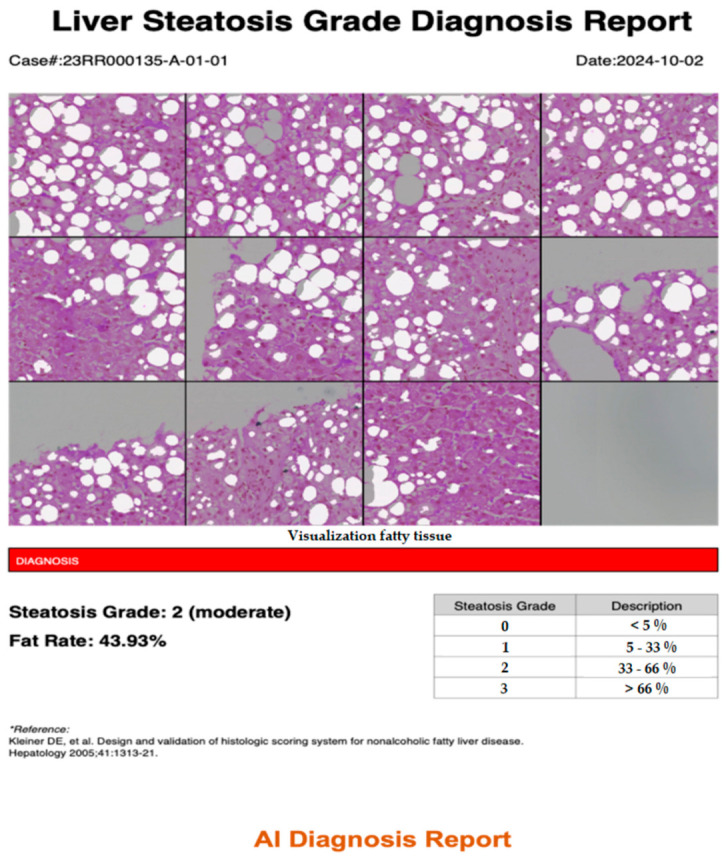
Sample report generated by SteatoStat. * Steatosis grading thresholds are derived from the histological scoring system proposed by Kleiner et al. (2005) [16].

SteatoStat can only fully realize its potential when it is fully integrated into the laboratory routine clinical workflow. As such, we have trialed the algorithm on clinical cases in our tertiary hospital laboratory without any significant delays to the reporting turn-around time. The sample report generated from SteatoStat comprising the segmented patch-level images, along with fat percentage and Steatosis grade, is represented in Figure 6. We are currently evaluating SteatoStat’s performance in the real-world setting, and hope that it can reduce inter- and intra-observer variability and subjectivity, allowing pathologists of varying expertise and experience to quantify fat in the liver accurately and better monitor patients’ conditions.

### 4.1. Limitations

(i) Lack of External Validation cohort

Despite the good performance of SteatoStat, we hope to validate our pipeline with an external cohort in the near future. As the calibration model was not evaluated using independent data splits, the reported agreement with pathologists and with the existing model represents internal validation. Incorporating external validation in future work will not only confirm the generalizability of the calibration approach and enhance the credibility of the model but also pave the way for its potential integration into real-world applications.

(ii) Relatively Lower DICE Scores of Other Liver Structures

While the overall DICE score for fat segmentation improved with the inclusion of multiple file formats and by using a foundational model like SAM, the performance gains were not uniform across all anatomical liver structures. In particular, some liver structures such as blood vessels demonstrated a relatively lower DICE score (Table 3), indicating challenges in accurately detecting and segmenting these regions. The reduced performance for certain anatomical structures is likely multifactorial, including class imbalance due to limited representation in the training data, increased intra-class variability, and reduced contrast between target structures and surrounding tissue. Furthermore, heterogeneity in WSI file formats may introduce compression artifacts and resolution inconsistencies, which can degrade feature representation and disproportionately impact the segmentation of smaller or morphologically complex structures. These findings highlight the need for more targeted preprocessing or augmentation strategies to ensure consistent segmentation performance across all relevant structures. With continued refinement and a more balanced representation of all the structures in the dataset, the model holds strong potential to achieve even greater segmentation accuracy across the board.

While the model performed well in the majority of cases, residual prediction errors, particularly in steatosis grades 1 and 2, highlight areas for improvement. These residual prediction errors may be attributed to data imbalances, inconsistent or subjective annotations, staining variability [26], and inadequate handling of tissue heterogeneity [27]. Additionally, possible model underfitting or overfitting [28] in regions of borderline morphology could have impacted performance. Future efforts should focus on improving annotation consistency, enhancing data quality through stain normalization [21], and ensuring balanced representation across steatosis grades [27]. Architectural enhancements such as attention mechanisms and uncertainty estimation [29] may improve performance in diagnostically ambiguous regions. Refinement strategies, including alternative loss functions, post hoc calibration [27], and ensemble learning [30], could further reduce prediction errors. Finally, incorporating enhanced feature engineering and domain-adaptive training will be important to improve generalizability and robustness across diverse histological presentations and institutional settings.

(iii) A current limitation of our work is that we did not evaluate the impact of varying patch sizes or sampling strategies. Exploring these design choices would be a valuable direction for future research, as it may offer deeper insight into the trade-off between preserving fine-grained local details and maintaining sufficient contextual information.

### 4.2. Future Direction

We have implemented SteatoStat within our high workload clinical anatomical laboratory in a tertiary hospital. In the future, we aim to use the same pipeline to trial quantification of hepatic steatosis in fresh frozen liver biopsy samples. We also hope to introduce quantification of other liver histological parameters such as fibrosis, ballooning degeneration and inflammation.

## 5. Conclusions

SteatoStat represents a significant advancement in the field of digital pathology by providing an automated, objective, and highly accurate framework for the grading of hepatic steatosis. By integrating various modules—ranging from file standardization to advanced segmentation models—the pipeline successfully translates complex liver structures into precise quantitative measurements and clinical grades. The high degree of reliability and agreement with expert pathologists demonstrates that SteatoStat is not only a suitable alternative to manual assessment but also a robust tool for standardizing steatosis quantification across clinical settings. Ultimately, this technology holds the potential to reduce variability in diagnosis and enhance the precision of liver disease management.

## Figures and Tables

**Table 2:** Average DICE score for fat based on image formats (U-Net) for a single split cohort.

Image Format		Mean DICE Score for Fat
Trained On	Tested On	
iSyntax	iSyntax	0.7000
		0.7859 (further refinement)
iSyntax	iSyntax	0.8540
	NDPI	0.5610
	iSyntax + NDPI	0.6700
iSyntax + NDPI	iSyntax + NDPI	0.8540
iSyntax + NDPI + iSyntax2	iSyntax + NDPI + iSyntax2	0.8955

**Table 3:** (**a**) Segmentation performance for a single split cohort—average DICE score of different liver structures (U-Net). (**b**) Overall segmentation accuracy via 5-fold cross-validation—average DICE score of different liver structures (U-Net versus SAM).

Structure/Model	Fat	Vessels	Collagen and Inflammation	Balloon Cell	Portal Tract	Central Vein	Nuclei
(**a**)							
Mean DICE score (U-Net)	0.8955	0.6000	0.5834	0.4583	0.6267	0.4285	0.5028
(**b**)							
Mean DICE score (U-Net)	0.7894	0.2456	0.7284	0.2593	0.6801	0.6929	0.6613
Mean DICE score (SAM)	0.8542	0.0971	0.4386	0.1773	0.4234	0.3392	0.33766

**Table 4:** AUROC and F1 performance metrics of SAM and U-Net segmentation models on fat.

Metric on Fat	SAM		U-Net	
	AUROC	F1	AUROC	F1
Mean	0.9928	0.8990	0.9562	0.7894

## Data Availability

The datasets generated during and/or analyzed during the current study are not publicly available due to patient privacy and ethical restrictions but are available from the corresponding author upon reasonable request.

## Supplementary Materials

The following supporting information can be downloaded at https://www.mdpi.com/article/10.3390/diagnostics16121825/s1. Figure S1: Strong correlation (r = 0.92) observed in the Pearson plot between pathologists’ median and SteatoStat’s predicted fat percentages; Figure S2: Bland–Altman plot showing strong agreement, with minimal bias (mean difference = 0.98) and predictions typically within ±20% (pathologists’ median fat percentage vs. SteatoStat’s predicted fat percentage); Figure S3: Boxplot analysis gradings showing a good concordance of distribution between predicted fat percentage and pathologists’ assigned steatosis grade; Figure S4: Bland–Altman plot showing moderate agreement with HistoIndex study, minimal bias (mean difference 1.17) and predictions typically within ±26%; Figure S5: Boxplot of a previous study’s steatosis gradings and SteatoStat’s predicted fat percentage; Table S1: (a) Performance metrics for each fold in the 5-fold cross-validation of fat segmentation model (SAM); (b) Performance metrics for each fold in the 5-fold cross-validation of fat segmentation model (U-Net); Table S2: Pathologists’ steatosis gradings of WSIs and their respective SteatoStat predicted fat percentages; Table S3: A previous study’s steatosis gradings of WSIs and their respective SteatoStat predicted fat percentages; Table S4: WSI-level dataset composition; Table S5: ROI distribution across annotation sources; Table S6: Patch-level dataset characteristics; Table S7: Scanner and file format distribution.

## Abbreviations

The following abbreviations are used in this manuscript:

MASLD	Metabolic Dysfunction-Associated Steatotic Liver Disease
NASH-CRN	Non-Alcoholic Steatohepatitis Clinical Research Network
AUROC	Area Under the Receiver Operating Characteristic Curve
SAM	Segment Anything Model
