# Supplementary material for: Real-World Insights in Designing SteatoStat: An End-to-End Deep Learning Pipeline for Hepatic Steatosis Quantification
Source: Diagnostics (Basel). 2026 Jun 12;16(12):1825. doi: 10.3390/diagnostics16121825 (PMC13298593; doi:10.3390/diagnostics16121825)
Supplement: Supplementary file 1 [file diagnostics-16-01825-s001.zip › diagnostics-4150339-supplementary.pdf]

SUPPLEMENTARY DATA

Table S1. (a) Performance metrics for each fold in the 5-fold cross validation of fat segmentation model (SAM); (b) Performance metrics for each fold in the 5-fold cross validation of fat segmentation model (U-Net).

Experiment	AUROC	F1	Precision	Recall
**(a)**				
Fat_1	0.993413092	0.924878636	0.957662603	0.894264981
Fat_2	0.993789003	0.84901284	0.910603395	0.795226079
Fat_3	0.992530318	0.922797903	0.950925255	0.896286701
Fat_4	0.988608328	0.882830666	0.916934352	0.851172859
Fat_5	0.995731155	0.915458879	0.946430649	0.886449966
**Mean**	**0.992814**	**0.898996**	**0.93651**	**0.86468**
**(b)**				
Fat_1	0.935789	0.805529	0.889692	0.738641
Fat_2	0.958883	0.776002	0.757993	0.797990
Fat_3	0.962948	0.842615	0.840384	0.847139
Fat_4	0.958366	0.706973	0.67892	0.803800
Fat_5	0.964996	0.815984	0.784861	0.858512
**Mean**	**0.956196**	**0.789421**	**0.790370**	**0.809216**

SteatoStat Correlation Analyses Against Pathologists

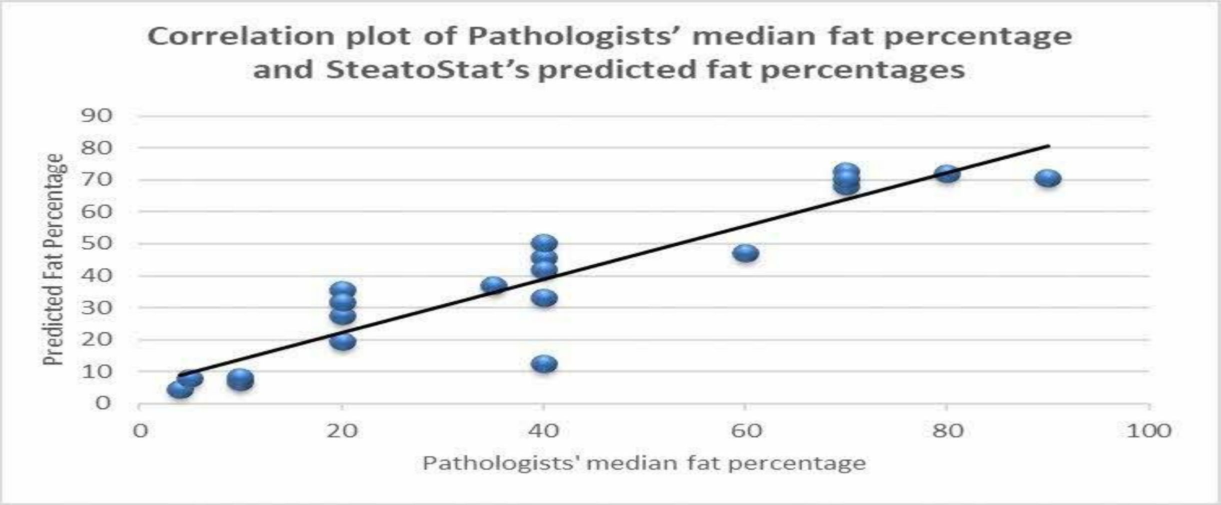

Figure S1. Strong correlation (r=0.92) observed in the Pearson plot between Pathologists' median and SteatoStat's predicted fat percentages.

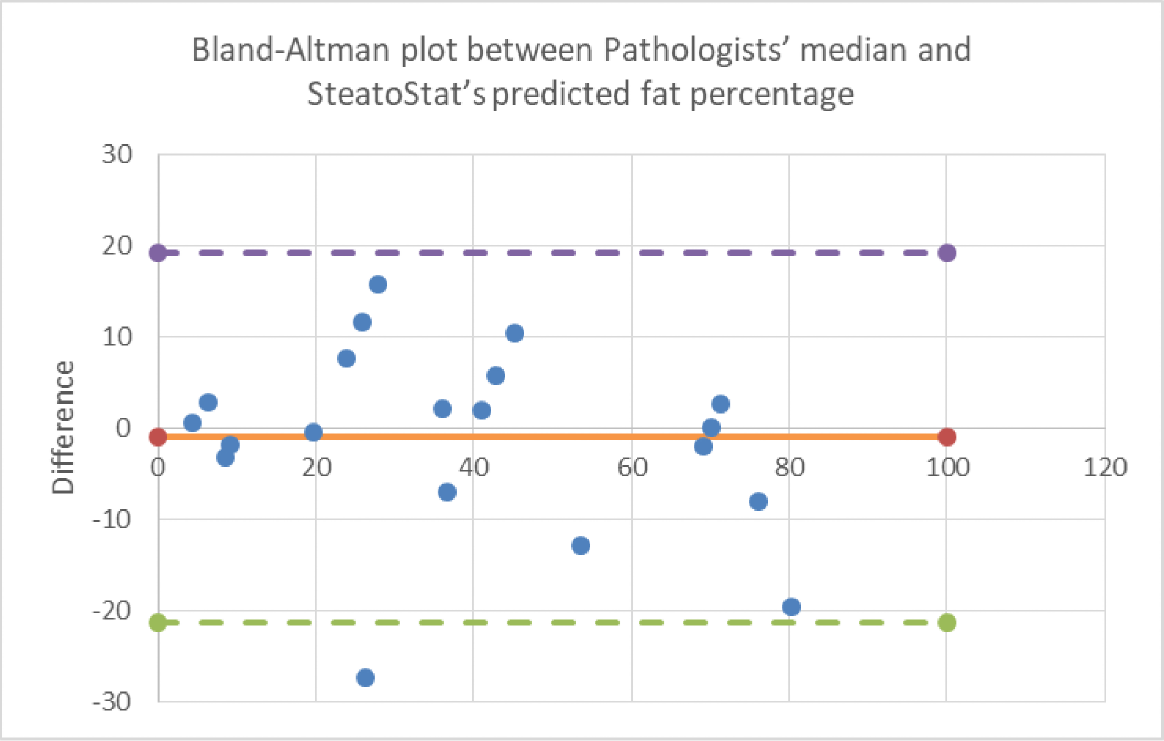

Figure S2. Bland-Altman plot showing strong agreement, with minimal bias (mean difference=0.98) and predictions typically within ±20% (Pathologists' median fat percentage vs SteatoStat's predicted fat percentage).

Mean absolute error and prediction accuracy

The MAE is also used to calculate the error without considering the direction of deviation of each individual value. The MAE is calculated as follows:

$$MAE = \frac{1}{n}\sum_{i=1}^{n}|y_i - \hat{y}_i|$$

where n is the total number of samples, denotes the predicted fat percentage and represents the ground truth fat percentage (pathologists' mean).[25] The fat percentage prediction accuracy is then calculated by the following formula:

$$\text{Fat prediction accuracy} = 100\% - [\left(\frac{Mean\ absolute\ error}{Mean\ ground\ truth}\right) \text{x } 100\%\ ]$$

The model achieved a mean absolute error (MAE) of 7.3% in predicted fat content and a fat prediction accuracy of 82%, indicating good overall performance in quantifying hepatic steatosis.

A Wilcoxon signed-rank test[26] was performed to compare predicted fat percentages between our model and the pathologists' median. The difference was not statistically significant (p = 0.97), indicating that there was no meaningful discrepancy between the two sets of predictions.

Performance evaluation on Fat scoring against Pathologists' grading

Distributional analysis by Box plot

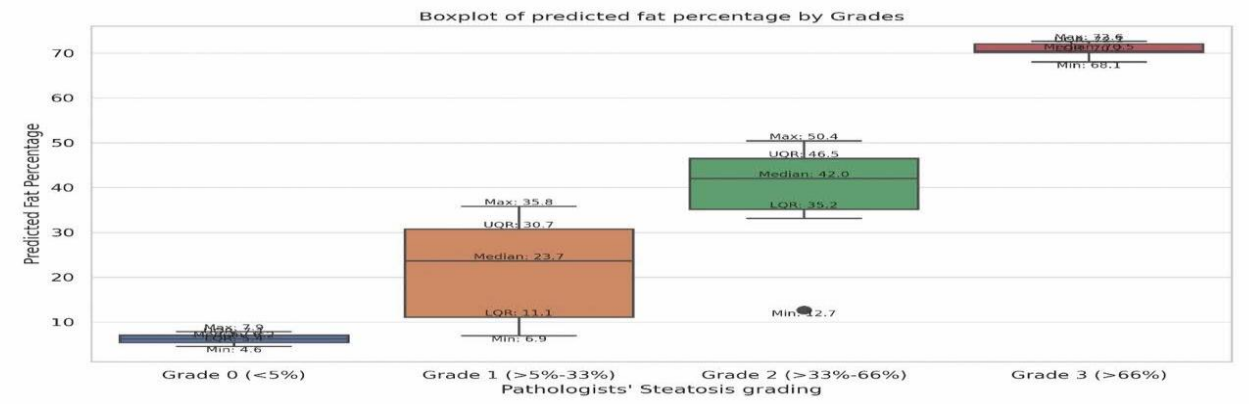

Figure S3. Boxplot analysis gradings showing a good concordance of distribution between predicted fat percentage and Pathologists' assigned steatosis grade.

Table S2. Pathologists' steatosis gradings of WSIs and their respective SteatoStat predicted fat percentages.

	**Grade 0 (<5%)**	**Grade 1 (>5%-33%)**	**Grade 2 (>33%-66%)**	**Grade 3 (>66%)**
1	4.592330	6.93540	45.76999	68.07845
2	7.874972	19.57954	37.17297	72.64996
3	NA	27.76084	12.67048	70.15231
4	NA	35.79379	50.43118	72.05582
5	NA	8.27928	33.14691	70.48277
6	NA	31.70569	47.16509	NA
7	NA	NA	42.04869	NA

For Grade 0 (steatosis <5%), predicted fat percentages were consistently low, with a median value below 5%. Grade 1 (>5–33%) showed wider variability, with predicted values ranging approximately from 6.9% to 35.8%, and a moderate interquartile spread, reflecting heterogeneity within this histologic category.

Grade 2 (>33–66%) demonstrated higher predicted fat percentages, with median values around 42–47% and relatively narrow interquartile ranges, suggesting good model performance in this intermediate range. Finally, Grade 3 (>66%) exhibited the highest predicted fat percentages (68–72%), with minimal overlap with lower grades, supporting the model's ability to differentiate advanced steatosis. Table S6 shows the raw values used to plot the boxplot.

SteatoStat Comparison Analyses against Histoindex[18]

Bland-Altman analysis:

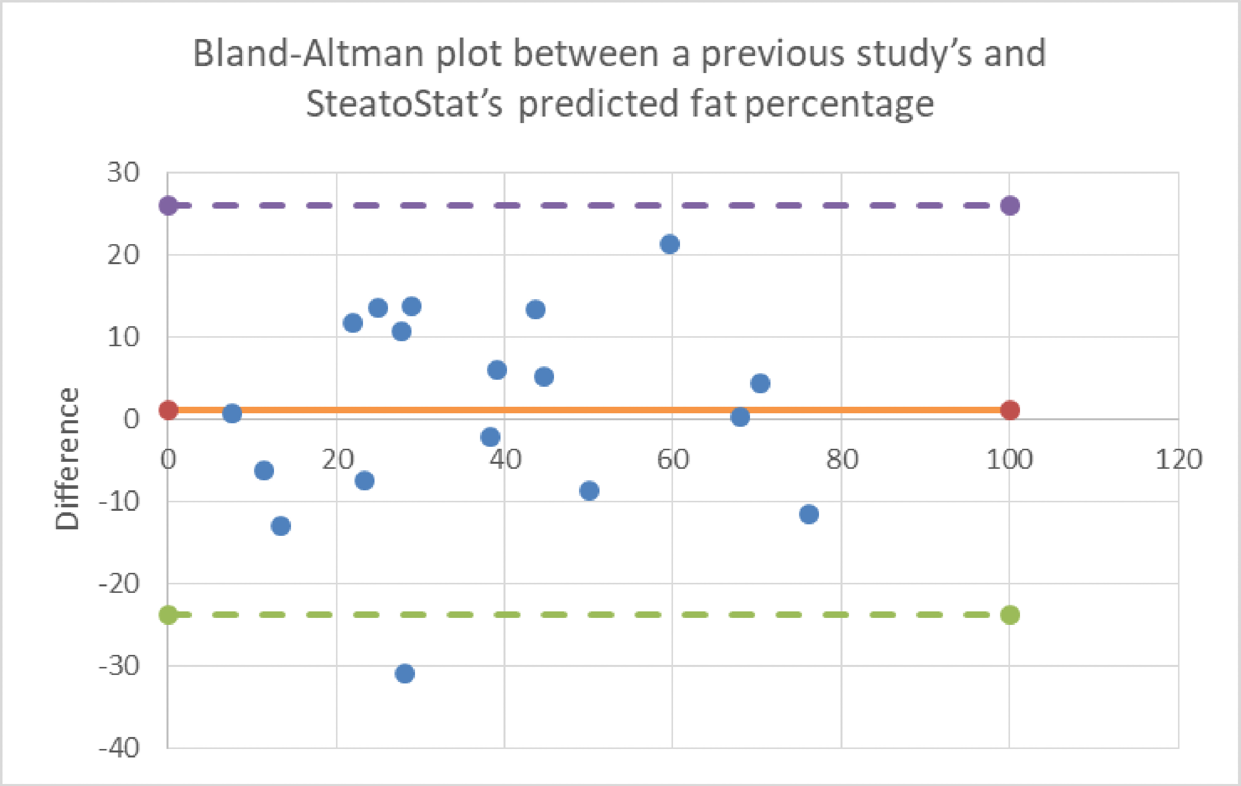

Figure S4. Bland-Altman plot showing moderate agreement with HistoIndex study, minimal bias (mean difference 1.17) and predictions typically within ±26%.

Mean absolute error and prediction accuracy

When compared with fat percentages predicted by Histoindex, our model achieved a **mean absolute error (MAE) of 9.11%** and a **prediction accuracy of 77%**, indicating good concordance in fat quantification performance.

The comparison between the two models using the Wilcoxon Signed-Rank Test[26] revealed no statistically significant difference in fat percentage predictions ($p = 0.45$), indicating that the two models provide comparable estimates.

Performance evaluation on Fat scoring against Histoindex grading

Distributional analysis by Box plot

In addition to categorical assessment, distributional analysis was done using the box plot. The plot (Figure 10) displays the distribution of model-predicted fat percentages stratified by histological steatosis grade (Grade 0–3) based on data from a prior study. A clear upward trend in predicted fat

percentage was observed with increasing histological grade, aligning with the expected biological pattern of hepatic steatosis progression.

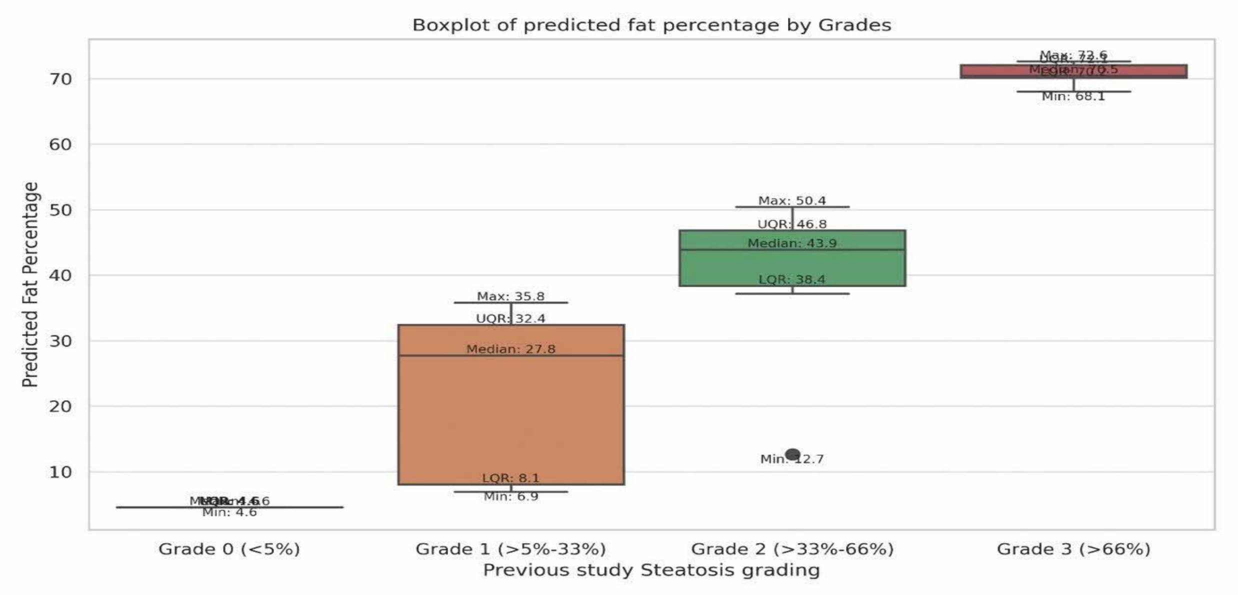

Figure S5. Boxplot of a previous study's steatosis gradings and SteatoStat's predicted fat percentage.

Table S3. A previous study's steatosis gradings of WSIs and their respective SteatoStat predicted fat percentages.

	Grade 0 (<5%)	Grade 1 (>5%-33%)	Grade 2 (>33%-66%)	Grade 3 (>66%)
1	4.59233	6.93540	45.76999	68.07845
2	NA	33.146908	37.17297	72.64996
3	NA	27.760839	12.67048	72.05582
4	NA	35.793785	50.43118	70.48277
5	NA	8.279280	47.16509	70.15231

6	NA	31.705690	42.04869	NA
7	NA	7.874972	NA	NA

For Grade 0 (<5%), the predicted fat percentage was low (median: ~4.6%), consistent with minimal steatosis. Grade 1 (>5–33%) showed a broader range (approximately 6.9% to 35.8%) with some variability, reflecting mild steatosis and heterogeneous fat content within this category.

Grade 2 (>33–66%) had higher predicted fat percentages (median range ~42–47%), with tighter clustering around the mid-range values, suggesting reliable prediction in moderate steatosis cases. Grade 3 (>66%) consistently showed the highest predicted fat values (median ~70–72%), with minimal overlap with lower grades. Table S7 shows the raw values used to plot the boxplot.

Table S4. WSI-level dataset composition.

Category	Count
Total WSIs	41
Patch-level annotated WSIs	9
WSI-level annotated WSIs	12
Subset for agreement analysis	20
5-fold cross-validation	41

Table S5. ROI distribution across annotation sources.

Source	ROIs
Patch-based (9 WSIs)	10,706
WSI-level annotations	1,448
**Total ROIs**	**12,154**

Table S6. Patch-level dataset characteristics.

Metric	Value
Total patches	90 (9 samples)
Patch size	1024 × 1024

Table S7. Scanner and file format distribution.

Format	Patches	Percentage
(Philips) iSyntax	6878	56.6
(Hamamatsu) NDPI	3828	31.5
(Philips) iSyntax2	1448	11.9
Total	12154	100
